# Deletions in chromosome 6p22.3-p24.3, including *ATXN1*, are associated with developmental delay and autism spectrum disorders

**DOI:** 10.1186/1755-8166-5-17

**Published:** 2012-04-05

**Authors:** Patrícia BS Celestino-Soper, Cindy Skinner, Richard Schroer, Patricia Eng, Jayant Shenai, Malgorzata MJ Nowaczyk, Deborah Terespolsky, Donna Cushing, Gayle S Patel, LaDonna Immken, Alecia Willis, Joanna Wiszniewska, Reuben Matalon, Jill A Rosenfeld, Roger E Stevenson, Sung-Hae L Kang, Sau Wai Cheung, Arthur L Beaudet, Pawel Stankiewicz

**Affiliations:** 1 Department of Molecular and Human Genetics, Baylor College of Medicine, Houston, TX, USA; 2 J.C. Self Research Institute of Human Genetics, Greenwood Genetic Center, Greenwood, SC, USA; 3 Neonatal-Perinatal Medicine, Pediatrics, The Vanderbilt Clinic, Nashville, TN, USA; 4 Pathology and Molecular Medicine and Pediatrics, Hamilton Regional Laboratory Medicine Program, Hamilton, ON, Canada; 5 Credit Valley Hospital, Mississauga, ON, Canada; 6 Specially for Children, Austin, TX, USA; 7 Division of General Academic Pediatrics, Department of Pediatrics, The University of Texas Medical Branch at Galveston, Galveston, TX, USA; 8 Signature Genomic Laboratories, PerkinElmer, Inc, Spokane, WA, USA

**Keywords:** 6p deletions, Copy-number variants, Array comparative genomic hybridization

## Abstract

Interstitial deletions of the short arm of chromosome 6 are rare and have been associated with developmental delay, hypotonia, congenital anomalies, and dysmorphic features. We used array comparative genomic hybridization in a South Carolina Autism Project (SCAP) cohort of 97 subjects with autism spectrum disorders (ASDs) and identified an ~ 5.4 Mb deletion on chromosome 6p22.3-p23 in a 15-year-old patient with intellectual disability and ASDs. Subsequent database queries revealed five additional individuals with overlapping submicroscopic deletions and presenting with developmental and speech delay, seizures, behavioral abnormalities, heart defects, and dysmorphic features. The deletion found in the SCAP patient harbors *ATXN1*, *DTNBP1*, *JARID2*, and *NHLRC1 *that we propose may be responsible for ASDs and developmental delay.

## Background

Deletions involving the distal part of the short arm of chromosome 6 are relatively rare. Terminal deletions of 6p24-pter have been associated with developmental delay, brain malformations (including Dandy-Walker malformation), anterior eye chamber abnormalities, hearing loss, ear abnormalities, micrognathia, and heart defects [1-6]. Patients with larger sized deletions of 6p23-pter also presented with microcephaly, genital anomalies, language impairment, and delayed motor development [1,3,5,7-14]. The identified ocular developmental abnormalities are caused by deficiency of the dosage sensitive *FOXC1 *gene (MIM 01090) [15-20]. In addition, deletions and duplications involving *FOXC1 *have been shown recently to be responsible for Dandy-Walker malformation [21].

Interstitial deletions of 6p22-p24 have been reported even less often and are generally associated with psychomotor and growth delay, hypotonia as well as several congenital abnormalities, including hydrocephalus, microcephaly, structural eye abnormalities, hypertelorism, low set and rotated ears, nasal anomalies, micrognathia, palatal abnormalities, short folded neck, defects of heart, kidney, and feet, abnormal genitalia, and abnormal fingers with hypoplastic nails [4,5,13,22-26].

Here, we describe six individuals, five of whom have overlapping interstitial deletions in chromosome 6p22.3-p24.3 encompassing *ATXN1*. The majority of patients had neurological or behavioral abnormalities, including developmental and speech delay, autism spectrum disorders (ASDs), attention deficit hyperactivity disorder (ADHD), repetitive behaviors, and various dysmorphic features.

### Clinical reports

#### Patient 1

**Figure 1:**
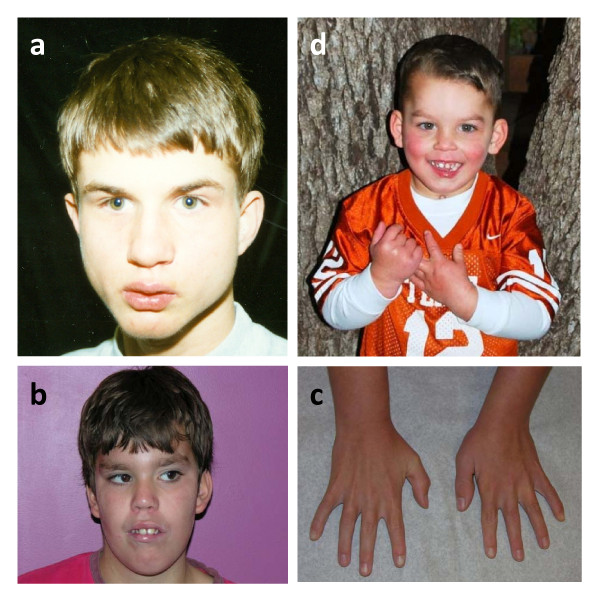
**Patients 1 (A), 4 (B-C), and 6 (D)**.

This 15-year-old male proband was enrolled in the South Carolina Autism Project (SCAP) study at the J.C. Self Research Institute of Human Genetics at the Greenwood Genetics Center in Greenwood, South Carolina. He was the second child of healthy parents. The pregnancy and delivery were uneventful. He was developmentally delayed, used a few words until 3 years of age, and remained nonverbal until 7-8 years of age. At 15 years of age, he used about six words but no phrases or sentences. The proband was also socially inappropriate and was classified in the autism spectrum according to ADOS and ADI-R testing. He had narrow facies, flat midface, overbite, slight prominence and jutting of the tissue of chin, and pits on the skin at the base of the nasal septum. His eyes appeared to be recessed due to flat midface, and his neck was long (Figure 1a). There were some flaring or winging of the scapulae. Wood's lamp exam revealed confluent minimally hypopigmented blotches about the size of a half dollar over his sacrum. Thumb tissues were somewhat broad distally. He had short thumbnails, broad and short feet, short and broad great toes, long second toes, and short third toes. Deep tendon reflexes were 1+ in the upper and lower limbs, and plantar reflexes were flexor. The father was unavailable for testing. This patient died at the age of 26 years following intestinal rupture.

#### Patient 2

This four-year-old male proband had developmental and speech delay, repetitive movements, hyperactivity, and possible ASDs; however, no formal ASDs testing was performed. Metabolic studies did not reveal any abnormalities. There were no dysmorphic features. A 2/6 systolic murmur was observed. Reportedly, the mother and the older brother of this patient are mentally disabled. DNA from the proband's older brother was not available.

#### Patient 3

This newborn female patient passed away soon after birth due to multiple congenital defects. The autopsy report described low set ears, bilateral partial aniridia, incomplete palpebral fissures bilaterally, micrognathia, shortened philtrum, redundant nuchal folds, two supernumerary nipples, hypoplastic toe nails, enlarged heart with multiple congenital defects (dilated right atrium, interrupted aortic arch, dilated pulmonary trunk with superiorly placed left pulmonary artery take-off, patent foramen ovale, two left and one right pulmonary veins draining to left atrium, perimembranous ventricular septal defect with portion of tricuspid valve extending through VSD, patent ductus arteriosus, and narrowed aortic and mitral valves), incomplete lobation of the right upper and middle lung lobes, bicornuate uterus, diastasis recti, bilateral simian creases of the hands, and anteriorly placed anus. The parents were not available for further studies.

#### Patient 4

This male patient was first seen at the age of six years. He was born by spontaneous vaginal delivery. His mother reported that the fetus was exposed to cigarette smoke and a large amount of alcohol. He presented with developmental delay, seizure disorder, attention defficit hyperactivity disorder (ADHD) diagnosed at 24 months, behavioral problems, anger and impulse control problems, speech problems, difficulties expressing himself, craniofacial dysmorphology, long face, almond shaped eyes, low set ears with forward facing lobules, thick malaligned philtrum, tall chin with retrognathia and overbite, pectus carinatum, toe abnormalities, and clubbed foot (Figure 1b, c). His father was described by the mother as having speech problems. Metabolic screening and fragile X testing of the proband were normal. Blood specimens from the parents were not available. He required right tibial and proximal fibular epiphysiodesis for leg length discrepancy at the age of 15. Currently, he is 17 years old with significant developmental delays and behavioral issues.

#### Patient 5

A 7-year-old-girl was described as being nondysmorphic and having developmental and speech delay, bilateral strabismus (corrected at four years of age), and three generalized seizures without fever at four months of age. Her EEG, MRI and CT were normal. The patient also had an ongoing iron deficiency, for which she was being treated. The mother had an uncomplicated pregnancy and an induced vaginal delivery. The parents are healthy, as are the older two full sisters and half sister.

#### Patient 6

This three-year-old male patient was born after an induced vaginal delivery due to maternal hypertension. There were no complications during the pregnancy. He was described as having stocky appearance, frontal upsweep, wide spaced and deep set eyes, broad forehead, mild micrognathia (status post tracheostomy), and small thumbs (Figure 1d). Other physical findings included biparietal bossing, small ears, narrow alveolar archis, missing teeth, tracheostomy scar, flared ribs, and bilateral clinodactyly. He had tiny frontal lobe hemorrhages on an MRI performed a few days after his birth. CT showed an arachnoid cyst. Additionally, he had hypotonia, a heart murmur, atrial septal defect, and bicuspid aortic valve, mandibular hypoplasia, a history of jaw distraction, heel cord release, asthma and recurrent aspiration pneumonia, a history of G-tube (removed), eczema, possible submucous cleft palate, speech problems, mild hearing loss on Auditory Brainstem Response Evaluation, fatigue, a history of febrile seizures, lack of coordination, and global developmental and learning delays. At the time of his visit, he was very hyperactive and did not speak any words. He was diagnosed with sensory processing disorder with developmental dyspraxia. The parents reported that he used two signs, had better receptive than expressive language (with a lot of body language), was easily distracted, destructive, and wild. Blood specimens from the parents were not available.

## Results

**Figure 2:**
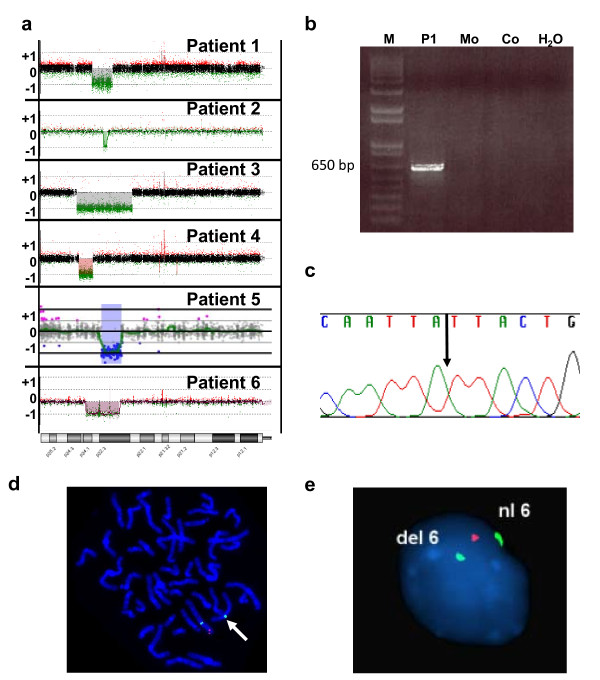
**Chromosome 6p deletion in patients 1-6 - a) Array CGH probe plots in patients 1-6**. X axis, chromosome 6 position; Y axis, log_2 _ratio. Semitransparent filled boxes on CGH plots highlight the region of aberration. **b**) Patient 1-specific junction fragment is not present in his unaffected mother (Mo) or unaffected control (Co). M, marker. **c**) Chromatogram showing the breakpoint fusion in the patient 1-specific junction fragment. **d**) Metaphase FISH showing a deletion at 6p22.3 in patient 5. Chromosome 6p22.3-specific BAC clone RP11-140A3 is labeled in red, and the chromosome 6 centromere control probe *D6Z1 *is labeled in green. The presence of one red signal indicates deletion of 6p22.3 on one homologue (arrow). **e**) Interphase FISH showing a deletion at 6p24.1-p22.3 in patient 6. Chromosome 6p23-specific BAC clone RP11-127P7 is labeled in red, and the chromosome 6 centromere probe is labeled in green as a control. The presence of one red signal indicates deletion on one homologue (del 6). Nl6, normal chromosome 6.

Using array comparative genomic hybridization (array CGH), in patient 1 with ASDs from the SCAP collection, we identified an ~ 5.4 Mb deletion on chromosome 6p22.3-p23, harboring 21 RefSeq genes (Figure 2a) and sequenced its breakpoints. The distal breakpoint (chr6:13,662,096) maps within a LINE element (L1MEe) and the proximal breakpoint (chr6:19,042,218) maps within a unique sequence. There was no microhomology between the deletion breakpoints (Figure 2c). G-banded chromosome slides were retrospectively reviewed at the Greenwood Genetics Center and an interstitial deletion 6p22.3p23 was detected (data not shown). Polymerase chain reaction (PCR) in the proband's mother showed that she was not a carrier for the deletion (Figure 2b).

Although the father of patient 1 was not available for analysis, we attempted to determine the deletion origin by CAG repeats polymorphism in *ATXN1*, a gene located within the deletion, and independently by SNP array analysis. The results of *ATXN1 *polyglutamine expansion were inconclusive (data not shown). SNP analysis showed that all 1,193 patient's SNPs in the deleted region (on the normal chromosome) matched the mother's SNPs for that region, and that 85% (33,541/39,459) of the patient SNPs in the non-deleted region of chromosome 6 matched the mother's SNPs. Thus, these results indicate that it is highly likely that the normal allele was inherited from the patient's mother and that the allele with the deletion arose on the paternal chromosome 6 (either inherited or *de novo*).

Array CGH images for deletions in patients 2-6 are shown in Figure 2a. Patient 2 had a 6p22.3 deletion of approximately 1 Mb that involves the *ATXN1*, *FLJ23152*, *RBM24*, and *CAP2 *genes. The deletion was confirmed by fluorescence in situ hybridization (FISH) analysis and was also found in the patient's mother, who was reported to have some degree of mental disability. Patient 3, who passed away soon after birth, had a deletion of approximately 14.6 Mb on chromosome 6p22.3-p24.3 that involves 58 RefSeq genes. Retrospective high-resolution partial karyotype analysis confirmed the interstitial deletion of 6p22.3-p24.3 (data not shown). Patient 4 had a deletion on chromosome 6p23-p24.3 of approximately 3.6 Mb that involves 28 RefSeq genes. Patient 5 had a 6p22.3 deletion of approximately 5.2 Mb that involves 18 RefSeq genes (Figure 2a, d). Parental FISH studies revealed that the deletion was *de novo*. Patient 6 had a deletion on chromosome bands 6p22.3-p24.1 of approximately 8.8 Mb that involves 34 RefSeq genes (Figure 2a, e). Patient 6 also had a 2.7 Mb gain on chromosome 15q11.2 (chr15:20,565,530-23,300,182), including the BP1-BP2 region, which has been observed in other probands and their normal parents and therefore most likely represents a familial copy-number variant.

### Deletion frequency in control populations

A query of the Database of Genomic Variants revealed no CNVs larger than 200 kb within the largest deletion reported in this study (patient 3). No exonic deletions involving *ATXN1 *were found in six control groups consisting of 2792 individuals [27], 2493 individuals [28], 2026 individuals [29], 1152 individuals [30], 450 individuals [31], and 270 individuals [32].

## Discussion

ASDs ([MIM 209850]) embody a group of behavioral abnormalities, including restricted and repetitive behaviors, and/or defects in social interaction and/or communication. Cytogenetic abnormalities were initially reported in 25-30% of individuals with ASDs and dysmorphic features [33], whereas pathological CNVs have been found in 5-10% of patients with milder ASDs through the use of oligonucleotide microarrays [34-38].

Using array CGH to search for CNVs that may be associated with ASDs in the patients from the SCAP collection, we identified a deletion at chromosome 6p22.3-p23 in a 15 year-old boy with ASDs. Further database [Medical Genetics Laboratories (MGL) and Signature Genomic Laboratories (SGL) diagnostic laboratories] and literature queries revealed additional five and 12 overlapping interstitial deletions, respectively. Patients 1, 2, and 4-6 had developmental and speech delays, features commonly seen in the published patients with the deletions in chromosome 6p22.3-p24.3 (Table 1). Patients 4-6 also had seizures, ADHD or hyperactivity, or repetitive behaviors. In addition, eight out of the 10 patients (pts A, C, E-H, J, and K) with interstitial deletions involving 6p22.3-p24.3 described in the literature (Table 1) had speech delay and one had hyperactivity. Patient 3, who passed away shortly after birth, and patients B and D [23,24], evaluated at 9 and 13 months of age, respectively, were too young to receive a diagnosis of ASDs and to display some of the behavioral abnormalities described above.

**Table 1:** Clinical features of 18 patients with interstitial deletions in 6p22-p24

Pt	Gender	Chr6 region	Coordinates (hg19)	Size (Mb)^(a)^	Inheritance	Age^(b)^	DD/ID	Speech delay	ASDs	Hyper activity/ADHD	Repetitive behavior	SZ	Hypotonia	CHD	Brain defect	Dysmorphic features^(c)^
1	M	p22.3-p23	13662096-19042218	5.4	NM	15 y	+	+	+	+	N/A	-	+	-	N/A	+

2	M	p22.3	16572367-17543199	1.0	M	4 y	+	+	+	+	+	-	-	(d)	N/A	-

3	F	p22.3-p24.3	9621501-24218259	14.6	UK	1 m	N/A	N/A	N/A	N/A	N/A	N/A	N/A	+	-	+

4	M	p23-p24.3	10269968-13915223	3.6	UK	17 y	+	+	-	+	+	+	-	-	-	+

5	F	p22.3	16186391-21421705	5.2	DN	7 y	+	+	-	N/A	N/A	+	-	N/A	N/A	-

6	M	p22.3-p24.1	12058814-20896726	8.8	UK	3 y	+	+	(e)	+	N/A	+	+	+	+	+

A	M	p22.2-p25.2 or p21.33-p23	(2.3-4.2) - (25.2-27.0) or (13.4-15.2) - (30.4-32.1)	N/A	DN	3 y	+	+	N/A	N/A	N/A	N/A	+	+	N/A	+

B	M	p22.3-p24	(7.1-13.4) - (15.2-25.2)	N/A	DN	9 m	+	N/A	N/A	N/A	N/A	N/A	N/A	+	+	+

C	M	p22.1/p22.2-p23	14.4 - 21.6	N/A	UK	15 y	+	+	N/A	(f)	N/A	N/A	+	N/A	N/A	+

D	F	p22.3-p23/p24.1	11.9 - 18.7	N/A	UK	13 m	+	N/A	N/A	N/A	N/A	N/A	+	+	+	+

E	F	p22.3-p24.1	(13.0-14.0) - 21.7	N/A	UK	34 m	+	+	N/A	N/A	N/A	N/A	+	+	+	+

F	M	p22.3-p24.1	10.0 - 15.8	N/A	NM	20 y	+	N/A	N/A	N/A	+ (g)	N/A	+	-	-	+

G	M	p22.3-p24.2	10.0 - 18.7	N/A	DN	4 y	+	+	N/A	N/A	N/A	N/A	N/A	-	+	+

H	M	p24.2-p25.1	(4.2-6.1) - 10.4-11.9)	N/A	DN	23 m	N/A	+	N/A	N/A	N/A	N/A	N/A	+	-	+

I	M	p23	13889301-15153952	1.3	DN	N/A	N/A	N/A	+	N/A	N/A	N/A	N/A	N/A	N/A	N/A

J	F	p22.1-p23	14446670-27741682	13.3	DN	16 y	+	+	N/A	+	N/A	N/A	N/A	+	-	+

K	F	p22.3	16132021-23152021	7.0	DN	4 y	+	+	-	-	N/A	N/A	-	+	-	+

L^(h)^	UK	p22.3	18829825-23576125	4.7	UK	N/A	+	N/A	+	N/A	N/A	N/A	+	N/A	N/A	+

**Total**							**15/15**	**12/12**	**4/8**	**5/7**	**3/3**	**3/5**	**8/12**	**9/14**	**5/11**	**15/17**

^a ^Minimum size in Mb.

^b ^Age at last clinical visit. Y, years old; m, moths old.

^c ^Dysmorphic features for patients 1-6 is are listed in Clinical Reports.

^d ^2/6 systolic murmur.

^e ^Sensory processing disorder with developmental dyspraxia.

^f ^Poor concentration

^g ^Behavioral problems included aggressiveness and tactile aversiveness.

^h ^DECIPHER declares that those who carried out the original analysis and collection of the data bear no responsibility for the further analysis or interpretation of it by the recipient or its registered users.

A, [25]; B, [24]; C, P1 from [23]; D, P2 from [23]; E, 91-145 from [4]; F, 95-800 from [4]; G, PF from [4]; H, [13]; I, AU010604 from [34]; J, [26]; K, [22]; L, DECIPHER patient ID 249613 [39]. +, feature present; -, feature absent; ASDs, autism spectrum disorders; CHD, congenic heart defect; DD, developmental delay; DN, *de novo*; F, female; ID, intellectual disability; M, male; M, maternal; NM, not maternal; SD, speech delay; SZ, seizures; N/A, information not available; Pt, patient identification; UK, unknown.

Patient 2 also presented with ASDs, suggesting that one or more loci within chromosome 6p22.3-24.3 may play a role in the development of ASDs. In support of this notion, a DECIPHER (Database of Chromosomal Imbalance and Phenotype in Humans Using Ensembl Resources) [39] patient (ID 249613) with an ~ 4.7 Mb interstitial deletion at chromosome 6p22.3 had autistic behavior in addition to hypotonia, developmental delay/intellectual disability, downslanting palpebral fissures, and strabismus (Table 1). Moreover, a patient with ASDs was reported to have a *de novo *~ 1.3 Mb interstitial deletion at 6p23 (Table 1) [34]. However, this patient also had a *de novo *2 Mb deletion in chromosome 13q14.12-q14.13.

Sixteen deletions (pts 2-6, A-G, and I-L) overlap with the deletion found in patient 1, who had a diagnosis of ASDs (Table 1). Thirteen (pts 2, 4-6, A, C, E-G, and I-L) out of those 16 patients (Table 1) had ASDs and/or some of the ASDs associated features: speech delay, ADHD or hyperactivity, or behavioral abnormalities [40,41]. We propose that some of the following genes may be responsible for the ASDs features and should be considered in further research testing in patients with ASDs: *ATXN1 *(deleted in 10 patients), *JARID2 *(deleted in eight patients), *DTNBP1 *(deleted in eight patients), and *NHLRC1 *(deleted in eight patients) (Table 2).

**Table 2:** Characterization of the selected genes on 6p22-p24

Gene Symbol	Function^a^	Disease Association	References	
			
			MIM	Entrez Gene ID
ATXN1*	RNA and protein binding; transcriptional repressor activity	SCA1 (MIM 164400)	601556	6310

*CAP2*	Actin binding	UK	N/A	10486

*CD83*	Immune response; signal transduction	UK	604534	9308

*CDKAL1*	Metal ion binding	Psoriasis (MIM 177900)	611259	54901

DTNBP1*	Organelle biogenesis; neuronal function	HPS (MIM 203300); schizophrenia (MIM 181500)	607145	84062

*E2F3*	DNA binding, transcription activator activity, control of cell cycle	UK	600427	1871

*FLJ23152*	UK (hypothetical protein)	UK	N/A	401236

*ID4*	Transcription repressor and co-repressor activity	UK	600581	3400

JARID2*	DNA, chromatin, and protein binding; transcription repressor activity; CNS development	UK	601594	3720

*MBOAT1*	Acetyltransferase activity, phospholipid biosynthesis	Dauwerse-Peters Syndrome (MIM 611733)	611732	154141

NHLRC1*	E3 ubiquitin ligase activity	MELF (MIM 254780)	608072	378884

*RBM24*	RNA and nucleotide binding	UK	N/A	221662

*RNF182*	Ubiquitin-protein ligase activity	UK	N/A	221687

^a ^Based on Gene Ontology Annotation [71].

* Strong candidate genes for autism or autistic features given their role in the brain function or development or involvement in neurological disorders.

CNS, central nervous system; HPS, Hermansky-Pudlak syndrome; MELF, myoclonic epilepsy of Lafora; N/A, not available; SCA1, spinocerebellar ataxia 1; UK, unknown.

Ten out of 13 patients with *ATXN1 *deletion (pts 1-3, 5, 6, A-E, G, J, and K) had ASDs, speech delay, ADHD or hyperactivity, or other behavioral abnormalities (Table 1). CAG trinucleotide extensions of 41-81 repeats within the coding region of *ATXN1 *are responsible for the autosomal dominant spinocerebellar ataxia 1 (SCA1 [MIM 164400]), a neurodegenerative disorder with progressive cerebellar degeneration. *ATXN1 *is also proposed to function as a regulator of gene expression [42]. Interestingly, *Atxn1 *homozygous knockout mice were shown to share aberrations with a knock-in mouse model of SCA1 that contained the polyglutamine extension [43,44]. Despite the fact that the knockout mice did not develop SCA1 or progressive cerebellar degeneration, both models had abnormalities in spatial learning and memory, motor learning and coordination, and in cerebellar gene expression [43,44]. Additionally, a meta-analysis has suggested that SNPs within *ATXN1 *are associated with intelligence quotient in the background of ADHD [45] and Bremer et al. [22] proposed that haploinsufficiency of *ATXN1 *may therefore contribute to the learning difficulties observed in the patients harboring a 6p22 deletion. Given its importance in brain function and behavior abnormalities in mouse models, we speculate that heterozygous deletions that affect *ATXN1 *function may be involved with the outcomes of developmental delay and ASDs, either alone or in combination with other gene deletions.

Homozygous missense point mutations in *DTNBP1 *have been found in patients with Hermansky-Pudlak syndrome (HPS [MIM 203300]), an autosomal recessive disease with features that include albinism, pulmonary fibrosis, and bleeding [46]. DTNBP1 has been proposed to function in organelle biogenesis [46], presynaptic glutamate release in rat neurons [47], neural plasticity in *Drosophila *[48], and to localize to axons in mouse cerebellum and hippocampus [49]. Additionally, a meta-analysis study found an association of SNPs in *DNTBP1 *with schizophrenia (SCZD [MIM 181500]) [50,51]. Finally, *DTNBP1 *has been connected with autistic features observed sometimes in patients with Duchenne muscular dystrophy (DMD [MIM 310200]) [52-54]. DTNBP1 binds to alpha and beta dystrobrevins, which compose the dystrophin-associated protein complex (DPC) [49].

*JARID2 *is expressed in embryonic and adult human neurons [55] and may function as a transcriptional repressor [56]; its mouse homolog *Jmj *is necessary for proper neural tube formation and cardiac development [57]. Recently, a significant association was found between a *JARID2 *SNP (rs7766973) and autism [58], making this gene another candidate for ASDs.

Homozygous deletions, insertions, missense, or nonsense mutations in *NHLRC1 *have been found in individuals with myoclonic epilepsy of Lafora (MELF [MIM 254780]), an autosomal recessive disorder characterized by rapid and progressive adolescent-onset epilepsy, mental and motor deterioration, and short survival. NHLRC1 functions as an E3 ubiquitin ligase that mediates degradation of EPM2A (or LAFORIN). Mutations that disturb EPM2A degradation, leading to the accumulation of Lafora bodies, are also causative for MELF [59].

**Figure 3:**
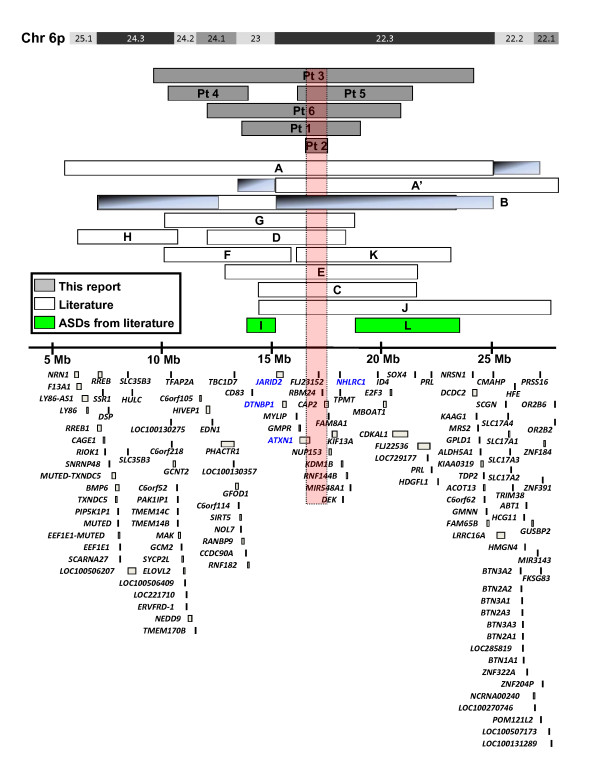
**Extent and gene (RefSeq) content of chromosome 6p22-p24 deletions**. Labeling of horizontal bars representing deletions described in the literature are as follows: **A**, [25]; **B**, [24]; **C**, P1 from [23]; **D**, P2 from [23]; **E**, 91-145 from [4]; **F**, 95-800 from [4]; **G**, PF from [4]; **H**, [13]; **I**, AU010604 from [34]; **J**, [26]; **K**, [22]; and **L**, DECIPHER patient ID 249613 [39]. Blue regions depict the maximum extents of the deletions. Longest gene isoforms are shown. Histone gene cluster on 6p22.1-p22.2 is not depicted due to space limitations. Gene names are positioned below or to the left of genes. Green bars represent individuals from the literature, who were reported to have ASDs. Gene names in blue represent strong candidates for ASDs or autistic features given their role in the brain function and development or involvement in neurological disorders (see Table 2). Red vertical bar depicts the smallest deletion overlap, involving 13 deletions (patients 1-3, 5, 6, A-E, G, J, and K). There are four RefSeq genes in this region: *ATXN1, FLJ23152*, *RBM24*, and *CAP*2. See Table 2 for gene functions. Pt, patient.

Although most individuals with deletions in chromosome 6p22.3-p24.3 display developmental delay, speech delay, and ASDs or other behavioral abnormalities, there is no single locus that is deleted in all patients with 6p22-p24 deletion (Figure 3). Penetrance of the phenotype caused by mutations in specific genes in this region is likely incomplete and may depend on the presence of modifiers found in the non-deleted alleles, regulatory regions, or other genes elsewhere in the genome. In addition, under-ascertainment with the lack of use of proper ASDs diagnostic tools such as ADOS and ADI-R may confuse the phenotype-genotype correlations.

Finally, four of the six patients described in this study had variable dysmorphic features, including craniofacial dysmorphisms, structural ear defects, and hands and feet abnormalities, which are commonly found in individuals described in the literature. Similar to the behavioral, developmental, and intellectual abnormalities described above, there is no single locus that is deleted in all individuals with 6p22-p24 deletion that may explain these dysmorphic features. Of note, other features that are commonly seen in patients described in the literature, such as congenital heart defects and hypotonia, were not found in all patients described in this report.

## Methods

### Consents

Written informed consents were approved by the Institutional Review Board of Self Regional Hospital at Greenwood, SC, for the J.C. Self Research Institute of Human Genetics (IRB 24), the Institutional Review Board for Human Subject Research at Baylor College of Medicine (H-25466), and by the Institutional Review Board of Spokane (study number 1500) and were obtained for patients 1, 4 and 6, and patient 5, respectively.

### Chromosomal microarray analysis

For patient 1, DNA was extracted from peripheral blood and lymphoblastoid cell line. DNA from the cell line was used for initial array CGH analysis at Baylor College of Medicine (BCM). DNA from peripheral blood was used to confirm the array CGH findings. For patients 2-4 and 6, DNA was extracted from whole blood using the Puregene DNA isolation kit (Gentra System, Inc., Minneapolis, MN). For patient 5, a blood specimen was sent to SGL, in Spokane, Washington for clinical array CGH analysis. Parental DNA was only tested by FISH.

All arrays used in this study were designed and analyzed based on genome build hg18 (NCBI Build 36, March 2006). The coordinates found in tables and figures were converted to hg19 (GRCh 37), February 2009 using UCSC liftOver [60].

Patient 1 was tested for copy-number variants (CNVs) using array CGH with an Agilent custom designed exon targeted microarray with coverage for 294 ASDs candidate genes (44 K, design ID 019729, Agilent Technologies, Inc., Santa Clara, CA) [61]. Patients 2-4 and 6 were studied using Agilent OLIGO custom clinical microarrays versions 7.4 OLIGO, 6.3 OLIGO, 6.4 OLIGO, and 8.0 OLIGO, respectively, designed at the MGL at BCM [62-65]. Patient 5 was studied using the 105 K-feature SignatureChip Oligo Solution^® ^whole-genome custom microarray manufactured by Agilent for SGL [66]. Additionally, the Agilent catalog SurePrint G3 Human CGH Microarray 1 × 1 M of design ID 021529 was used to confirm CNVs in patients 1, 3, and 4.

The protocols for DNA digestion, labeling, purification, hybridization, array scan, and analysis of the clinical arrays followed the manufacturer's instructions with some modifications as previously described [63,66,67].

### SNP array analysis

SNP genotyping for chromosome 6 was done in patient 1 and his mother using the commercially available Illumina Human 610-Quad BeadChip Kit (Illumina, Inc., San Diego, CA). Arrays were scanned using the Illumina Iscan with Autoloader2. SNP genotyping and absence of heterozygosity analyses were performed using the Illumina GenomeStudio data analysis software.

### FISH analysis

Confirmatory FISH analyses were performed in patients 2 and 4-6 using standard cytogenetic procedures with bacterial artificial chromosome (BAC) clones RP11-346 F18, RP11-637019, RP11-140A3, and RP11-127P7, respectively. The parental samples were tested for patients 2 and 5.

### PCR and sequence analysis

PCR to confirm the 6p22.3-p23 deletion and to amplify the junction fragment in patient 1 was performed using Takara LA PCR kit (Takara Bio, Inc., Shiga, Japan) with the forward primer 5' - TGGTGTAGTTAAGGGGAAAGAGAGAGGAG - 3' and the reverse primer 5' - CTGCAGTATAAGCATACTACTACCCACTTAGGG - 3' (Sigma-Aldrich Corp., St. Louis, MO). To test the patient's mother for a low level somatic mosaicism, which might have been missed by the array CGH assay, PCR was performed using GoTaq^® ^Flexi DNA Polymerase kit (Promega Corporation, Madison, WI) with the forward primer 5' - TTTGGATTGGGAGGAATGAA - 3' and reverse primer 5' - GGGGAAAGAAACGGAACATC - 3' (Sigma-Aldrich Corp.). PCR products were analyzed using 1% agarose gel electrophoresis, purified from the agarose gel using the PCR Purification Kit (Qiagen, Valencia, CA), and then sent for Sanger dideoxy sequencing (SeqWright and Lone Star, Houston, TX).

### *ATXN1 *(*ATAXIN1*) STR extension analysis

To determine the size of the polyglutamine track in the *ATXN1 *gene, we followed the previously published procedures with minor modifications [68-70]. PCR was performed using Applied Biosystems Amplitaq's PCR kit (Applied Biosystems, Foster City, CA) with forward primer 5' - AACTGGAAATGTGGACGTAC - 3' and reverse primer 5' - CAACATGGGCAGTCTGAG - 3' (Integrated DNA Technologies, Inc., Coralville, IA). PCR products were analyzed by electrophoresis on a sequencing polyacrylamide gel. The gel was visualized by autoradiography at -70°C overnight.

## Abbreviations

ADHD: Attention deficit hyperactivity disorder; ASDs: Autism spectrum disorders; BAC: Bacterial artificial chromosome; BCM: Baylor College of Medicine; CGH: Comparative genomic hybridization; CNV: Copy-number variant; DECIPHER: Database of chromosomal imbalance and phenotype in humans using ensembl resources; FISH: Fluorescence in situ hybridization; MGL: Medical Genetics Laboratories; PCR: Polymerase chain reaction; SCAP: South Carolina autism project; SGL: Signature Genomic Laboratories.

## Competing interests

Many of the authors are faculty members in the Department of Molecular and Human Genetics at BCM, which offers extensive genetic laboratory testing including use of arrays for genomic copy-number analysis, and the Department derives revenue from this activity. JAR is an employee of Signature Genomics, a subsidiary of PerkinElmer, Inc.

## Authors' contributions

ALB and PBSC-S designed the experiments. PBSC-S and PE performed the chromosomal microarray analysis. PBSC-S, AW, and JW performed the SNP and *ATXN1 *STR extension analysis. PBSC-S and PS performed the PCR and sequence analysis. JAR, S-HLK, SWC, and PS signed out the microarray-based hybridization analysis results and analyzed FISH results. CS, RS, JS, MMJN, DT, DC, GSP, LI, RM, and RES clinically examined the patients. PBSC-S and PS drafted the manuscript. All authors read and approved the final manuscript.
